# A critical re-analysis of cases of post-transplantation recurrence in genetic nephrotic syndrome

**DOI:** 10.1007/s00467-021-05134-4

**Published:** 2021-05-24

**Authors:** Anna E. Mason, Moin A. Saleem, Agnieszka Bierzynska

**Affiliations:** Bristol Renal, Translational Health Sciences, Bristol Medical School, University of Bristol, Dorothy Hodgkin Building, Whitson Street, Bristol, BS1 3NY UK

**Keywords:** Nephrotic syndrome, Post-transplantation disease recurrence, Genetic

## Abstract

**Background:**

Genetic defects in podocyte proteins account for up to 30% of steroid-resistant nephrotic syndrome (SRNS) in the paediatric population. Most children with genetic SRNS are resistant to immunosuppression and at high risk of progression to stage 5 chronic kidney disease. Kidney transplantation is often the treatment of choice. The possibility of post-transplantation disease recurrence in genetic SRNS remains controversial, and poses fundamental questions about disease biology.

**Methods:**

We critically evaluated the published cases of post-transplantation recurrence in genetic patients, particularly testing ‘mutations’ against the most recent population variant databases, in order to clarify the diagnoses, and compare the clinical courses and responses to therapy.

**Results:**

Biallelic pathogenic variants in *NPHS1* leading to a complete absence of nephrin were the most commonly reported and best understood instance of nephrotic syndrome occurring post-transplantation. This is an immune-mediated process driven by antibody production against the novel nephrin protein in the allograft. We also identified a number of plausible reported cases of post-transplantation recurrence involving pathogenic variants in *NPHS2* (8 patients, biallelic), one in *WT1* (monoallelic) and one in *NUP93* (biallelic). However, the mechanism for recurrence in these cases remains unclear. Other instances of recurrence in genetic disease were difficult to interpret due to differing clinical criteria, inclusion of patients without true pathogenic variants or the influence of other factors on renal outcome.

**Conclusions:**

Overall, post-transplantation recurrence remains very rare in patients with genetic SRNS. It appears to occur later after transplantation than in other patients and usually responds well to plasmapheresis with a good renal outcome.

**Supplementary Information:**

The online version contains supplementary material available at 10.1007/s00467-021-05134-4.

## Introduction

Nephrotic syndrome (NS) has an estimated annual incidence of 2–5 per 100,000 children and is characterised by proteinuria, oedema and hypoalbuminaemia. Eighty percent of children are steroid responsive and subsequently have a good long-term prognosis. The remainder are steroid resistant, either from presentation (primary steroid resistance) or following an initial period of steroid sensitivity (secondary steroid resistance). Those with steroid-resistant nephrotic syndrome (SRNS) are at risk of extrarenal complications and 30–40% develop stage 5 chronic kidney disease (CKD 5) within 10 years of follow-up. Focal segmental glomerulosclerosis (FSGS) is the commonest renal histology in SRNS. The podocyte cell is crucial in FSGS pathogenesis with disruption to the glomerular filtration barrier, via podocyte injury, leading to loss of permselectivity and albuminuria.

Steroid-sensitive nephrotic syndrome (SSNS) and a subset of SRNS cases are considered to be immunologically mediated and a proportion are caused by an unidentified proteinuric circulating factor(s) produced due to T cell dysfunction. Evidence for the presence of a circulating factor includes the post-transplantation recurrence of proteinuria in SRNS patients and the increased glomerular permeability to albumin caused by FSGS patient serum in rat glomeruli in vitro [1, 2]. Possibly the remainder of SRNS cases are due to genetic disorders, leading to structural defects in the slit diaphragm and podocyte cytoskeletal components, although some may be due to other as yet unclear mechanisms. Over 70 genes are currently associated with NS and have been shown to account for up to 30% of SRNS cases, including the majority of those presenting within the first year of life [3–17].

Those with a genetic defect have a different pathophysiology, clinical course and response to therapy than those with immune-mediated disease. Most are resistant to immunosuppression, progress more rapidly to CKD 5 and have a lower risk of recurrence following transplantation [3, 18]. Kidney transplantation is often the treatment of choice in this group of patients and knowledge regarding the likely post-transplant clinical course is important to counsel families effectively.

Recurrence of nephrotic syndrome after transplantation is common, occurring in 30–50% of FSGS cases after the first transplant and up to 80% at subsequent transplantations [19]. The onset of proteinuria is generally early (median time 14 days) and shows some response to plasmapheresis and intensified immunosuppression, such as cyclophosphamide [19]. However, despite this treatment response, FSGS recurrence continues to have a negative impact on graft survival with significant physical, social and psychological consequences. The pathogenesis of post-transplantation recurrence remains uncertain but is thought to share a common aetiology with idiopathic FSGS, where circulating plasma factor(s) are postulated to act on the podocyte and disrupt glomerular permeability — so called circulating factor disease. Much work has focused on identifying risk factors for recurrence in order to guide transplantation decisions. Factors which influence recurrence risk include age at diagnosis, rate of progression to CKD 5 and original biopsy result [19, 20]. Paediatric patients with secondary steroid resistance have a much higher post-transplantation recurrence risk than those who were steroid resistant from presentation [21]. However, by far the most important factor to take into consideration is the underlying aetiology of disease. In general, patients with a genetic cause of NS do not recur after transplantation due to correction of the underlying defect and transplantation is therefore the treatment of choice [22]. However, there are a few exceptions to this rule which clinicians should be aware of. Most common is post-transplantation nephrotic syndrome occurring in patients with biallelic pathogenic variants in *NPHS1* leading to an absence of nephrin. This occurs due to the production of antibodies against the novel nephrin protein in the allograft so should be considered as ‘anti-nephrin’ antibody disease, rather than disease recurrence [23].

The question of whether recurrence of NS post-transplantation can occur in genetic NS, and whether this recurrence has the same characteristics as circulating factor disease remains controversial, and poses fundamental questions about the biology of recurrent disease. This report will focus on the reported cases of post-transplantation recurrence in patients with Mendelian NS with a particular focus on re-analysing the genetic variants reported, in the light of current population-level data on rare variants that allows us to assign pathogenicity far more accurately than has been historically possible.

## Methods

**Fig. 1:**
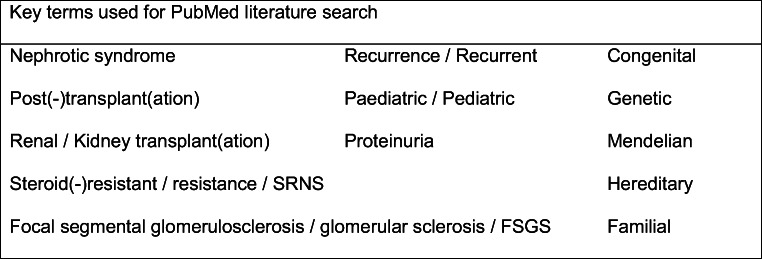
Key terms used for the PubMed literature search. All currently known genes associated with NS were also used as search terms [3–17]

Reported cases of post-transplantation recurrence in genetic NS were identified from the literature using a PubMed search and the authors’ knowledge of the topic. Key terms used in the PubMed search criteria are shown in Fig. 1. Further cases were then identified using citation chasing; analysing the bibliography of references for each paper (backward citation chasing) and through Google Scholar (forward citation chasing).

**Fig. 2:**
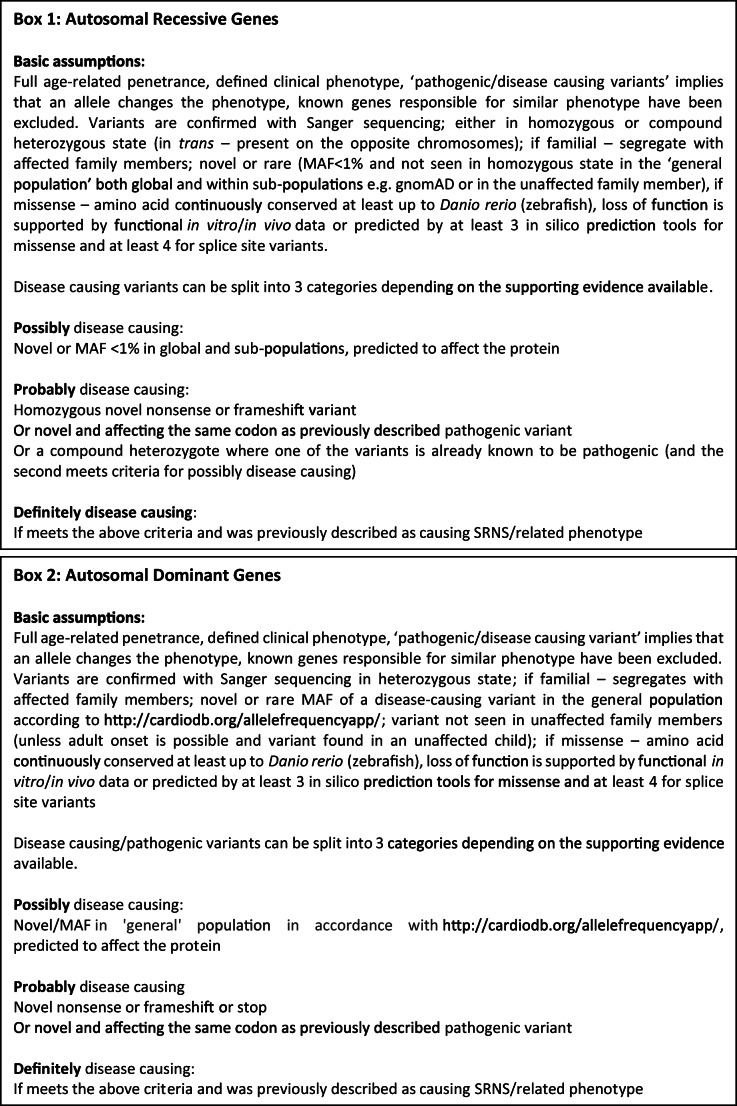
Criteria used to identify patients with true pathogenic variants in recessive (Box 1) or dominant (Box 2) genes. MAF, minor allele frequency

Each reported case was reviewed by the authors and the genetic variants re-analysed in view of the current population-level data now available for rare variants. The criteria used to identify true pathogenic variants are shown in Fig. 2. Standard clinical criteria for NS recurrence were used [24]. If further genetic or clinical information was required, the corresponding authors were contacted.

## Results of a critical review of reported cases of genetic post-transplant recurrence

### *NPHS1* and the formation of antibodies

Biallelic pathogenic variants in *NPHS1* are the most commonly reported and best understood instance of post-transplantation disease in genetic nephrotic syndrome. *NPHS1* encodes nephrin, a type-1 transmembrane protein found at the podocyte slit diaphragm, and mutations in this gene are responsible for most cases of congenital nephrotic syndrome (CNS). Fin-major c.121_122delCT; p.(Leu41Aspfs*50) and Fin-minor c.3325C>T; p.(Arg1109*) account for the majority of Finnish-type CNS (78% and 16% respectively), but 373 other *NPHS1* likely pathogenic variants have also been identified to date (HGMD® Professional 2020.4). Homozygous Fin-major is a well-known pathogenic variant leading to the complete absence of nephrin in the native kidney. Post-transplantation disease in these patients is an immune-mediated process driven by the production of antibodies against the novel nephrin protein in the allograft. One study of patients with *NPHS1* Finnish-type CNS demonstrated post-transplantation nephrotic syndrome in 25% with a mean time to disease onset of 12 months [23]. All patients with post-transplantation disease had the homozygous Fin-major variant and anti-nephrin antibodies were detected in 60%. Immunosuppressive therapy led to remission in some cases, but loss of function occurred in almost half of allografts. Antibody production can explain the pathogenesis of post-transplantation nephrotic syndrome in *NPHS1*-associated NS, which is most accurately considered as ‘anti-nephrin’ antibody disease rather than true disease recurrence. However, the cause is less clear in the few reported cases of recurrence associated with other genetic defects.

### NPHS2

Currently, there is little definitive evidence that SRNS patients with defects in genes other than *NPHS1* will experience post-transplantation recurrence. However, there are a few reports that suggest this can occur in association with certain *NPHS2* biallelic pathogenic variants. Pathogenic variants in *NPHS2,* encoding podocin, have an autosomal recessive pattern of inheritance and account for the majority of infantile and childhood-onset SRNS. Podocin, a raft-associated membrane component located at the insertion of the slit diaphragm, maintains the stability of the slit diaphragm network through recruitment of nephrin and CD2AP to microdomains. Podocin null mice have been shown to develop extensive proteinuria, podocyte foot process effacement and interestingly lack nephrin as well as podocin [25]. This slit diaphragm disassembly in the absence of podocin was also shown by Huber et al. through cell transfection studies [26]. More than 200 pathogenic *NPHS2* variants have now been identified (HGMD® Professional 2020.4) and are associated with a wide spectrum of clinical phenotype. For instance, the p.(Arg138GIn), which is the most common *NPHS2* pathogenic variant, manifests as very early-onset disease [27]. Conversely, the p.(Arg229Gln) non-neutral polymorphism can lead to late-onset NS when found in association with specific pathogenic *NPHS2* variants [28]. Despite the large number of pathogenic variants found in podocin, post-transplantation recurrence has been described in only a few patients with true biallelic pathogenic variants (Table 1, patients 1–8) [18, 27, 29–32, 35]. Other reported cases of recurrence involve patients with single heterozygous variants which, given the autosomal recessive inheritance of *NPHS2*, should not be considered to have genetic NS (Table 2). This will be discussed in more detail later.

**Table 1:** Reported cases of post-transplantation recurrence of nephrotic syndrome in patients with true pathogenic variants (pathogenicity criteria defined in Fig. 2). Recurrence is deemed ‘possible’ when it occurred late (> 3 months) and/or there was insufficient information available. Accession numbers; *NPHS2* NM_014625.4, *NUP93* NM_014669.4, *WT1* NM_024426.6. Allele count and MAF (minor allele frequency; gnomAD v2.1.1 The Genome Aggregation Database, http://gnomad.broadinstitute.org/). Allele count = allele count/ number of homozygotes/allele number). The MAF is taken from the total/global population. Nephrotic-range proteinuria is defined in children as > 40 mg/m^2^/h or uPCR > 2000 mg/g (Kidney disease improving global outcomes, KDIGO guidelines). *CP* cyclophosphamide. *CsA* cyclosporin A. *d* days. *FSGS* focal segmental glomerulosclerosis. *FU* follow-up post-transplantation. *H* homozygous. *m* months. *MP* methylprednisolone. *uPCR* urine protein creatinine ratio. *PP* plasmapheresis. *w* weeks. *y* years

Patient	Gene	Pathogenic variant	Allele count	MAF	Age at onset	Age at ransplant	Time to recurrence	Proteinuria at recurrence	Histology at recurrence	Likelihood of disease recurrence	Treatment	Outcome	Reference
1	*NPHS2*	c.413G>A:p.Arg138Gln **(H)**	163/0/282690	0.0005766	11m	9y	10d	300 mg/m^2^/h	–	Probable	PP (15 cycles) CP (2mg/Kg) 60d	Good	Bertelli 2003 [29]
2	*NPHS2*	c.413G>A:p.Arg138Gln **(H)**	163/0/282690	0.0005766	23m	4.5y	300d	120 mg/m^2^/h	FSGS	Possible	PP (6 cycles) CP (4 pulses 1g)	Good	Bertelli 2003 [29]
3	*NPHS2*	c.948delT: p.Ala317Leufs*31 **(H)**	4/0/282410	0.00001416	8m	4.5y	7d	uPCR 2400 mg/g	–	Probable	MP (500mg 3d) Increased CsA Ramipril 3.75mg/d	Good	Billing 2004 [30]
4	*NPHS2*	c.412C>T:p.Arg138* **(H)**	4/0/251298	0.00001592	2m	3y	4y	uPCR 5500 mg/g	FSGS, mesangial proliferation	Possible	PP (11w)	Slow reduction in graft function	Becker-Cohen 2007 [31]
5	*NPHS2*	c.412C>T:p.Arg138* **(H)**	4/0/251298	0.00001592	–	–	2y	–	FSGS Tacrolimus toxicity	Possible	–	–	Weber 2004 [27]
6	*NPHS2*	c.378G>T:p.Lys126Asn c.948delT: p.Ala317Leufs*31	- 4/0/282410	- 0.00001416	0m	6.6y	–	–	–	Possible	–	–	Ruf 2004 [18]
7	*NPHS2*	c.419del: p.Gly140Aspfs*41 **(H)**	2/0/251358	0.000007957	19m	–	–	–	–	Possible	–	–	Caridi 2003 [32]
8	*NPHS2*	c.538G>A:p.Val180Met c.467dupT: p.Leu156Phefs*11	3/0/250804 42/0/204742	0.00001196 0.0002051	96m	–	–	–	–	Possible	–	–	Caridi 2003 [32]
9	*NUP93*	c.1772G>T:p.Gly591Val c.1916T>C:p.Leu639Pro	38/0/281912 –	0.0001348 –	‘Infantile’	6y	1.7y	uPCR 4460 mg/g	Subtotal fusion of podocytes, early FSGS	Possible	PP Rituximab	Good (3.1y FU)	Seeman 2018 [33]
10	*WT1*	c.1447+4C>T:p.? (aka IVS9+4C>T)	–	–	6y	11y	7d	> 40 mg/m^2^/hr	NA	Probable	PP (9 cycles)	Good (3y FU)	Ghiggeri 2006 [34]

**Table 2:** Reported cases of post-transplantation recurrence of nephrotic syndrome in patients with single heterozygous variants (in recessive genes), and therefore unlikely true genetic cause. Accession numbers; *CD2AP* NM_012120.3, *NPHS2* NM_014625.4. Allele count and MAF (minor allele frequency; gnomAD v2.1.1 The Genome Aggregation Database, http://gnomad.broadinstitute.org/). Allele count = allele count/ number of homozygotes/allele number). The MAF is taken from the total/global population. Nephrotic-range proteinuria is defined in children as > 40 mg/m^2^/h or uPCR > 2000 mg/g (Kidney disease improving global outcomes, KDIGO guidelines). *ATN* acute tubular necrosis. *CP* cyclophosphamide. *CsA* cyclosporin A. *d* days. *CKD 5* stage 5 chronic kidney disease. *FSGS* focal segmental glomerulosclerosis. *FU* follow-up post-transplantation. *m* months. *MP* methylprednisolone. *PD* peritoneal dialysis. *uPCR* urine protein creatinine ratio. *PP* plasmapheresis. *w* weeks. *y* years

Patient	Gene	Variant	Allele count	MAF	Age at onset	Age at transplant	Time to recurrence	Proteinuria at recurrence	Histology at recurrence	Treatment	Outcome	Reference
11	*NPHS2* *CD2AP*	c.622G>A:p.Ala208Thr c.1488G>T:p.Met496Ile	13/0/251248 32/0/250916	0.00005174 0.000003985	3.5y	8.5y	2w	–	ATN, FSGS	PP	Pre-terminal kidney failure (1.5y FU)	Lowik 2008 [36]
12	*NPHS2*	c.59C>T:p.Pro20Leu	363/2/120298	0.003018	52m	133m	20d	240 mg/m^2^/hr	FSGS	PP (18 cycles) CP (4 pulses 1g)	CKD 5-PD after 3m	Bertelli 2003 Caridi 2003 [29, 32]
13	*NPHS2*	c.59C>T:p.Pro20Leu	363/2/120298	0.003018	117m	180m	10d	120 mg/m^2^/hr	–	PP (20 cycles)	Good	Bertelli 2003 Caridi 2003 [29, 32]
14	*NPHS2*	c.631T>A:p.Ser211Thr	1/0/251252	0.000003980	366m	512m	25d	200 mg/m^2^/hr	–	PP (8 cycles)	2^nd^ recurrence after 1m	Bertelli 2003 [29]
15	*NPHS2*	c.976dupA: p.Thr326Asnfs*20	–	–	–	–	18d	–	FSGS (at 1y)	–	Loss of allograft after 4y	Weber 2004 [27]
16	*NPHS2*	c.59C>T:p.Pro20Leu	363/2/120298	0.003018	–	–	3m	‘massive’	–	PP Tacrolimus	–	Weber 2004 [27]
17	*NPHS2*	c.709G>C:p.Glu237Gln	208/2/282220	0.0007370	–	–	18m	–	–	PP IV CsA	–	Weber 2004 [27]

Anti-podocin antibodies have never been identified [29, 31]. This may be because podocin consists of a hairpin membrane domain with two intracellular ends but no extracellular domain. It is therefore a ‘hidden’ protein which may not trigger antibody production. In addition, antibodies should only be stimulated following transplantation when the original pathogenic variant led to truncation or absence of the protein, such as the p.(Arg138*) which results in podocin truncation. However, many pathogenic variants in *NPHS2* are missense which lead to the production of whole protein and the allograft podocin would not be expected to trigger antibody production in these cases. Post-transplantation recurrence in *NPHS2*-associated NS therefore suggests that there must be an alternative aetiology for recurrence. The possible underlying mechanisms for this will be explored in more detail later.

### WT1

One case of post-transplantation recurrence in genetic NS has been reported in association with the Wilms tumour suppressor gene 1 (*WT1*) which fulfils our pathogenic variant criteria (Table 1, patient 10). *WT1* encodes a zinc finger transcription factor involved in kidney and gonadal development. *WT1* may affect podocyte structural proteins as it transcriptionally activates the *NPHS1* promoter, leading to upregulation of nephrin mRNA. Pathogenic variants in this gene (autosomal dominant mode of inheritance — one copy of the gene affected) usually result in syndromic forms of nephrotic syndrome, namely Frasier and Denys–Drash syndrome, but have also been associated with isolated SRNS. This patient developed nephrotic syndrome and FSGS on biopsy in association with Frasier syndrome (triad of glomerulopathy, male pseudohermaphroditism and gonadoblastoma) [34]. She had a pathogenic *WT1* splice site variant c.1447+4C>T:p.? (aka IVS9+4C>T), and received a deceased-donor transplant at 11 years of age. Seven days after transplantation, she developed proteinuria, which gradually increased to nephrotic levels. This responded well to plasmapheresis and allograft function remained good after three years of follow-up. There is a second report of recurrence in a patient with a de novo heterozygous c.1399C>T:p.Arg467Trp (aka p.Arg394Trp) pathogenic variant in exon 9 of the *WT1* gene and early-onset nephrotic syndrome associated with Denys–Drash syndrome [37]. However, this patient had evidence of immune-complex glomerulonephritis on biopsy indicating a different pathogenesis of disease (Table 3, patient 19).

**Table 3:** Reported cases of post-transplantation recurrence of nephrotic syndrome in genetic patients where there is insufficient clinical evidence to confirm disease recurrence, or other significant contributory factors to the clinical picture. Accession numbers; *ACTN4* NM_004923.6, *NPHS2* NM_014625.4, *WT1* NM_024426.6. Allele count and MAF (minor allele frequency; gnomAD v2.1.1The Genome Aggregation Database, http://gnomad.broadinstitute.org/). Allele count = allele count/ number of homozygotes/allele number). The MAF is taken from the total/global population. Nephrotic range proteinuria is defined in children as > 40 mg/m^2^/h or uPCR > 2000 mg/g (Kidney disease improving global outcomes, KDIGO guidelines). *CNI* calcineurin inhibitor. *CsA* cyclosporin A. *d* days. *FSGS* focal segmental glomerulosclerosis. *FU* follow-up post-transplantation. *GN* glomerulonephritis. *m* months. *MPGN* membranoproliferative glomerulonephritis. *uPCR* urine protein creatinine ratio. *PP* plasmapheresis. *SRL* sirolimus. *w* weeks. *y* years

Patient	Gene	Pathogenic variant	Allele count	MAF	Age at onset	Age at transplant	Time to recurrence	Proteinuria at recurrence	Histology at recurrence	Treatment	Outcome	Notes	Reference
18	*NPHS2*	c.413G>A:p.Arg138Gln c.535-1G>T:p.?	163/0/282690 –	0.0005766 –	2m	7y	10y	445 mg/m^2^/hr (10.7 g/m^2^/d)	FSGS, chronic CNI-induced nephrotoxicity	Conversion back to CsA	Good (2.5y FU)	Recurrence followed switch from CsA to SRL	Hocker 2006 [35]
19	*WT1*	c.1399C>T:p.Arg467Trp (aka p.Arg394Trp)	–	–	4y	5y	4y	uPCR 350 mg/g (35 g/mol)	Immune complex GN with an MPGN pattern	No changes made	Stable uPCR 1840 mg/g (184 g/mol) (8y FU)	Proteinuria not nephrotic range, biopsy suggests different disease pathogenesis	Neuhaus 2011 [37]
20	*ACTN4*	c.175T>C: p.Trp59Arg	–	–	5y	10y	2y	uPCR 1900 mg/g	Non-specific interstitial fibrosis and tubular atrophy. FSGS not excluded	PP	NA	Proteinuria not nephrotic range	Weins 2005 [38]

### NUP93

There is also one published case of post-transplantation recurrence in a patient with compound heterozygous pathogenic variant in nucleoporin 93 (*NUP93).* This is a relatively newly identified cause of genetic NS with onset in early childhood. NUP93 is an essential component of the nuclear pore complex (NPC), which regulates the transport of protein and RNA between the cytoplasm and the nucleoplasm. Knockdown of *NUP93* has been shown to impair NPC assembly, cell migration, proliferation and SMAD signalling pathways. The reported patient had a compound heterozygous variant affecting exons 16 and 18 of *NUP93* (c.1172G>T and c.1916T>C). He developed infantile SRNS, with biopsy-proven FSGS, and received a deceased-donor transplant at 6 years of age (Table 1, patient 9) [33]. Nephrotic range proteinuria (4460 mg/g) occurred 1.7 years post-transplant, triggered by an upper respiratory tract infection, with early signs of FSGS recurrence on biopsy. The proteinuria responded well to plasmapheresis, but became plasmapheresis-dependent. Rituximab was introduced with good effect and the patient remains proteinuria negative 3.1 years after transplant.

## Analysis of clinical course of recurrence

Despite aggressive treatment protocols, recurrence remains a significant cause of allograft loss and paediatric patients with FSGS have a lower allograft survival rate when compared to patients with other primary diseases. However, the clinical picture is variable as some patients with recurrent proteinuria maintain adequate kidney function for several years. Post-transplantation recurrence of NS usually occurs early, with a median time to proteinuria of 14 days [19]. It is interesting to note that four patients with genetic NS, meeting our pathogenic variant criteria, suffered post-transplantation recurrence much later than would be expected (Table 1, patients 2, 4, 5 and 9; 300 days, 4 years, 2 years and 1.7 years, respectively). The majority of patients responded quickly to plasmapheresis and/or immunosuppression and had good graft function. This may suggest that recurrence in genetic NS is a different entity to other FSGS recurrence, with a potentially better clinical course and outcome. Further evaluation of the time course, treatment response and long-term outcome will be needed from future reports of recurrence in genetic NS before this theory can be determined.

## Discussion

The potential for, and characteristics of, post-transplantation recurrence in genetic NS is currently controversial and has implications both for our understanding of disease biology and our ability to make transplantation decisions. There is now a greater degree of population-level data available on rare variants allowing us to assign pathogenicity far more accurately than before. In light of this, we have reviewed and summarised all the published cases of recurrence in genetic NS according to genetic and clinical re-appraisal (Tables 1, 2 and 3). This provides a clearer view on the risk and characteristics of recurrence in patients with true genetic NS which will help to guide clinical management.

Finn major in *NPHS1* remains the most well-established cause of post-transplantation nephrotic syndrome due to ‘anti-nephrin’ antibody disease. However, there is limited evidence that patients with other genetic causes of NS will recur following transplantation. Following re-analysis of the genetic variants, we identified cases of plausible post-transplant recurrence in eight patients with true pathogenic variants in *NPHS2*, one in *WT1* and one in *NUP93* that met our pathogenicity criteria. The mechanism for post-transplant disease is clear in *NPHS1*-associated NS, where antibodies are produced against the nephrin protein in the allograft. However, the pathogenesis of recurrence in other forms of genetic NS remains uncertain. No anti-podocin antibodies have been detected in any tested patient, even in patients with truncating variants [29, 31]. This may be due to difficulties in antibody detection techniques. However, several factors suggest that antibodies may not be the cause: (1) there is no evidence of immunoglobulin deposition in the allograft [29, 31], (2) the onset of recurrence is very early [31] and (3) discrete focal FSGS lesions are seen on biopsy whereas auto-antibodies would be expected to produce a more diffuse pattern of injury [29]. It is thought that one or more circulating plasma factors are present which alter glomerular permeability to proteins and cause proteinuria [1]. This factor(s) remains so far unidentified but its production appears to follow T cell dysfunction. Serum from patients with FSGS increases rat glomerular permeability to albumin in vitro [2]. Measuring volume changes of these rat glomeruli on exposure to FSGS serum has been used as an indirect measure of permeability activity (P_alb_). High P_alb_ values are predictive of post-transplantation recurrence in FSGS [39]. In one study, the risk of recurrence in patients with a positive proteinuric factor was ten times greater than in those where it was undetectable [39]. Carraro et al*.* identified two patients with *NPHS2-*associated NS, meeting our pathogenic variant criteria, and high P_alb_ values pre-transplant and at the time of recurrence (Table 1, patients 1–2) [40]. In one patient, P_alb_ values directly correlated with proteinuria and decreased progressively with plasmapheresis. The good clinical response to plasmapheresis and/or immunosuppression in post-transplantation recurrence is additional support for the role of a systemic factor in this process. Permeability activity may be determined by the balance of plasma factors that either induce or inhibit permeability. Apolipoproteins have been shown to inhibit permeability induced by FSGS serum in vitro [41]. Loss of inhibitory serum components in the urine during proteinuria may play an important role in the recurrence of FSGS post-transplantation [42].

Whilst post-transplantation recurrence generally occurs early (median 14 days), it is interesting that four of these 10 genetic NS patients had a much later onset of recurrence than would be expected (300 days, 1.7 years, 2 years and 4 years). The majority of patients also responded quickly to treatment and had good graft function, raising the possibility that recurrence in genetic NS is a different entity than other FSGS recurrence. However, further evaluation of the time course, treatment response and long-term outcome in future reports of recurrence in genetic NS will be needed to explore this further. Whilst late recurrence may be a clue to a different type of disease recurrence in genetic NS, it is also possible that these cases represent chronic allograft nephropathy rather than true recurrence.

Apart from these 10 cases, the other reported instances of recurrence in genetic disease were difficult to interpret due to differing clinical criteria, inclusion of patients without true pathogenic variants or the influence of other factors on renal outcome. Interpreting these as definite evidence for post-transplantation recurrence in genetic NS would over-estimate the risk in this patient group. Some studies include patients with single heterozygous variants (Table 2). Pathogenic variants in genes, such as *NPHS2,* with an autosomal recessive pattern of inheritance need a variant on both alleles (biallelic) in order to be disease-causing. Single heterozygous variants (found on only one allele) should therefore not be considered pathogenic. It is possible that the development of proteinuria in carriers of single heterozygous podocin variants is due to multigenic inheritance, with the presence of an additional pathogenic variant which has not yet been discovered. However, it is more likely that these patients have non-genetic NS caused by circulating factor disease and experience post-transplantation recurrence in the same way as other patients in this group. Clear criteria should be followed to identify and include only patients with true pathogenic variants and give a more accurate risk of post-transplantation recurrence in this group (Fig. 2).

There is also variability in the clinical criteria used to define post-transplantation recurrence. The reported case of post-transplantation recurrence in a child with α-actinin-4 gene (*ACTN4*)-associated NS (autosomal dominant) is one example where the clinical details are unconvincing of true recurrence (Table 3, patient 20) [38]. The affected child had a de novo p.(Trp59Arg) heterozygous pathogenic variant and received a living-related donor transplant from his father (normal *ACTN4* genotype) at 10 years of age. He developed recurrence of proteinuria post-transplantation but this was of a low level (urine protein creatinine ratio 1900 mg/g). Two years later, his graft function declined and a biopsy was taken. Although recurrent FSGS could not be excluded, the biopsy was not clearly indicative of recurrence showing non-specific interstitial fibrosis with tubular atrophy. The clinical evidence to confirm true recurrence in this case is limited. No other cases of recurrence in *ACTN4-*associated NS have been reported to date and treating this clinical scenario as evidence that it can occur may be misleading when counselling other families with the same condition.

Some episodes of recurrence reported in the literature may actually represent secondary FSGS due to post-transplant morbidity, such as rejection, reflux nephropathy, uncontrolled hypertension or immune-mediated glomerulonephritis [31]. One example of this is a case of post-transplantation recurrence reported in a patient with a heterozygous pathogenic variant in an autosomal dominant gene – *INF2* (inverted formin 2) [43]. We contacted the lead author who subsequently feels this was secondary FSGS due to renal scarring as a result of multiple post-transplant kidney injuries. Side-effects of immunosuppression may also contribute to the development of proteinuria following transplantation. One case is reported of a patient with a pathogenic compound heterozygous *NPHS2* variant (p.Arg138Gin + c.535-1G>T) who developed biopsy-proven FSGS recurrence ten years following transplantation (Table 3, patient 18) [35]. This coincided with a medication change from cyclosporin A to sirolimus. Conversion back to the original regimen led to significant improvement in proteinuria and graft function. It may be that sirolimus was insufficient to suppress the immune activation, thereby inducing proteinuria [44]. Cyclosporin A has been shown to reduce proteinuria in non-immune diseases through several mechanisms and, of particular interest in NS, stabilises podocyte foot processes through increased expression of synaptopodin [45]. An overview of the reported cases of genetic NS with insufficient clinical evidence to support true disease recurrence, or other significant contributing factors, can be seen in Table 3. Including cases such as these, which may not signify true recurrence, may lead to an overestimate of the risk. Clear clinical criteria to define post-transplantation disease recurrence should be used consistently throughout the literature. As a minimum, this should include the de novo development of nephrotic range proteinuria without a plausible alternative clinical scenario. In those cases where there is doubt, foot process effacement on biopsy and prompt response to intensified immunosuppression (including plasma exchange) are strongly supportive features.

In addition to the effect of recipient genotype on the risk of post-transplantation recurrence, several donor-dependent genetic factors have been identified which may influence long-term allograft survival. A form of post-transplant recurrence, not related to circulating factors, is of a living donor (LD) carrying a genetic risk allele in the donor kidney. There has already been much discussion in the literature about the use of LD kidney transplants in patients with genetic NS. In summary, in cases of autosomal recessive inheritance, a parent who was a heterozygous carrier would be accepted as a LD due to the negligible recurrence risk, except for carriers of pathogenic variants in *COL4A* genes [24]. Another potential exception is the podocin p.(Arg229Gln) non-neutral polymorphism which encodes a protein with altered nephrin-binding capability when compared to wild-type podocin [28]. This polymorphism increases the risk of adult-onset FSGS when associated with another pathogenic *NPHS2* variant. In contrast, if there is an identified pathogenic variant in an autosomal dominant gene, or a family history suggestive of dominant inheritance, living-related donation should not be used. The exception would be if donor genetic testing for the identified causative variant could be carried out prior to transplantation. Autosomal dominant pathogenic variants can present with variable penetrance and phenotype, including adult-onset NS. Therefore, even if the donor does not have evidence of disease, LD transplant may increase the risk of NS in the recipient and the donor.

**Fig. 3:**
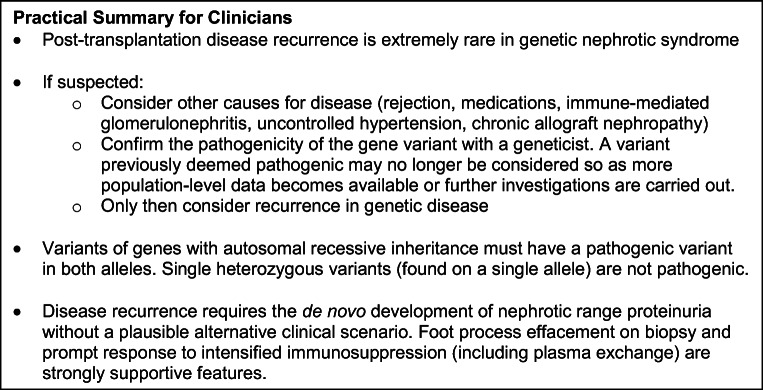
Key information and practical advice for clinicians

A practical summary for clinicians can be seen in Fig. 3. In conclusion, post-transplantation recurrence remains very rare in patients with genetic NS. Whilst some convincing cases do exist, the wider literature should be interpreted with caution due to the differing genetic or clinical criteria, or the influence of other factors on renal outcome. Clear criteria should be followed to identify and include only patients with true pathogenic variants and give a more accurate risk of post-transplantation recurrence in this group. Post-transplantation recurrence in genetic NS appears to occur later, and have better graft outcomes, than in other patients and this warrants further investigation.

## Supplementary Information

ESM 1 (DOCX 23 kb)

## Data Availability

Data sharing is not applicable to this article as no datasets were generated during the current study.
