# Supplementary material for: A critical re-analysis of cases of post-transplantation recurrence in genetic nephrotic syndrome
Source: Pediatr Nephrol. 2021 May 24;36(11):3757–69. doi: 10.1007/s00467-021-05134-4 (PMC8497325; doi:10.1007/s00467-021-05134-4)
Supplement: Supplementary file 1 — (DOCX 23 kb) [file 467_2021_5134_MOESM1_ESM.docx]

|  |  | **Minor Allele Frequency within Different Sub-Populations of gnomAD** | | | | | | | | |
| --- | --- | --- | --- | --- | --- | --- | --- | --- | --- | --- |
| **Gene** | **Variant** | **Total Population** | **African/African-American** | **Ashkenazi Jewish** | **East Asian** | **European (Finnish)** | **European (non-Finnish)** | **Latino/Admixed American** | **Other** | **South Asian** |
| **True Pathogenic Variants** | | | | | | | | | | |
| ***ACTN4*** | c.175T>C:p.Trp59Arg **(h)** | - | - | - | - | - | - | - | - | - |
| ***NPHS2*** | c.378G>T:p.Lys126Asn **(H, C)** | - | - | - | - | - | - | - | - | - |
|  | c.412C>T:p.Arg138* **(C)** | 0.00001592 | 0.00006163 | 0 | 0 | 0 | 0.00001760 | 0.00002893 | 0 | 0 |
|  | c.413G>A:p.Arg138Gln **(H, C)** | 0.0005766 | 0.0001604 | 0 | 0 | 0.00007962 | 0.001147 | 0.00002823 | 0.0008317 | 0.00006533 |
|  | c.419del:p.Gly140Aspfs*41 **(H)** | 0.000007957 | 0 | 0 | 0 | 0 | 0.00001759 | 0 | 0 | 0 |
|  | c.467dupT:p.Leu156Phefs*11 **(C)** | 0.0002051 | 0.0005724 | 0 | 0.0001895 | 0.0001573 | 0.0002210 | 0.0001608 | 0.0001885 | 0.0001534 |
|  | c.535-1G>T:p.? **(C)** | - | - | - | - | - | - | - | - | - |
|  | c.538G>A:p.Val180Met **(C)** | 0.00001196 | 0 | 0 | 0 | 0 | 0.000008831 | 0.00002896 | 0 | 0.00003267 |
|  | c.948delT:p.Ala317Leufs*31 **(H, C)** | 0.00001416 | 0.00004006 | 0 | 0 | 0 | 0.00002329 | 0 | 0 | 0 |
| ***NUP93*** | c.1772G>T:p.Gly591Val **(C)** | 0.0001348 | 0 | 0 | 0 | 0 | 0.0002950 | 0 | 0 | 0 |
|  | c.1916T>C:p.Leu639Pro **(C)** | - | - | - | - | - | - | - | - | - |
| ***WT1*** | c.1399C>T:p.Arg467Trp  (aka c.1180C>T:p.Arg394Trp) **(h)** | - | - | - | - | - | - | - | - | - |
|  | c.1447+4C>T:p.?  (aka IVS9+4C>T) **(h)** | - | - | - | - | - | - | - | - | - |
| **Not Pathogenic** | | | | | | | | | | |
| ***NPHS2*** | c.59C>T:p.Pro20Leu **(S)** | 0.003018 | 0.01013 | 0.0002198 | 0 | 0.0003332 | 0.001969 | 0.002543 | 0.003228 | 0.007006 |
|  | c.622G>A:p.Ala208Thr **(S)** | 0.00005174 | 0 | 0 | 0 | 0 | 0.00008802 | 0.00002894 | 0.0003268 | 0 |
|  | c.631T>A:p.Ser211Thr **(S)** | 0.000003980 | 0 | 0 | 0 | 0 | 0.000008802 | 0 | 0 | 0 |
|  | c.709G>C:p.Glu237Gln **(S)** | 0.0007370 | 0.00004008 | 0.009753 | 0 | 0 | 0.0005901 | 0.0002263 | 0.002366 | 0.0001635 |
|  | c.976dupA:p.Thr326Asnfs*20 **(S)** | - | - | - | - | - | - | - | - | - |
| ***CD2AP*** | c.1488G>T:p.Met496Ile **(S)** | 0.000003985 | 0 | 0 | 0.00005451 | 0 | 0 | 0 | 0 | 0 |

**Supplementary Table 1: Minor allele frequencies (MAF) of the genetic variants in the reported cases of post-transplantation recurrence in genetic nephrotic syndrome.**

MAF are taken from gnomAD v2.1.1 (The Genome Aggregation Database, <http://gnomad.broadinstitute.org/>). Pathogenic variants must have an MAF <1% (equivalent to <0.01) in both the total and the sub-populations (full pathogenicity criteria are described in Figure 2). *ACTN4* and *WT1* are autosomal dominant genes. *CD2AP, NPHS2* and *NUP93* are autosomal recessive genes. The variants deemed not pathogenic occurred in these patients as single heterozygous variants in an autosomal recessive gene. Accession numbers; *ACTN4* NM_004923.6, *CD2AP* NM_012120.3, *NPHS2* NM_014625.4, *NUP93* NM_014669.4, *WT1* NM_024426.6. C = compound heterozygous, H = homozygous, h = heterozygous, S = single heterozygous.
